# Spatially regulated editing of genetic information within a neuron

**DOI:** 10.1093/nar/gkaa172

**Published:** 2020-03-23

**Authors:** Isabel C Vallecillo-Viejo, Noa Liscovitch-Brauer, Juan F Diaz Quiroz, Maria F Montiel-Gonzalez, Sonya E Nemes, Kavita J Rangan, Simon R Levinson, Eli Eisenberg, Joshua J C Rosenthal

**Affiliations:** 1 The Eugene Bell Center, Marine Biological Laboratory, 7 MBL Street, Woods Hole, MA 02540, USA; 2 Raymond and Beverly Sackler School of Physics and Astronomy, Tel Aviv University, Tel Aviv 69978, Israel; 3 Department of Physiology and Biophysics, University of Colorado at Denver, Anschutz Medical Campus, Aurora, CO 80045, USA

## Abstract

In eukaryotic cells, with the exception of the specialized genomes of mitochondria and plastids, all genetic information is sequestered within the nucleus. This arrangement imposes constraints on how the information can be tailored for different cellular regions, particularly in cells with complex morphologies like neurons. Although messenger RNAs (mRNAs), and the proteins that they encode, can be differentially sorted between cellular regions, the information itself does not change. RNA editing by adenosine deamination can alter the genome’s blueprint by recoding mRNAs; however, this process too is thought to be restricted to the nucleus. In this work, we show that ADAR2 (adenosine deaminase that acts on RNA), an RNA editing enzyme, is expressed outside of the nucleus in squid neurons. Furthermore, purified axoplasm exhibits adenosine-to-inosine activity and can specifically edit adenosines in a known substrate. Finally, a transcriptome-wide analysis of RNA editing reveals that tens of thousands of editing sites (>70% of all sites) are edited more extensively in the squid giant axon than in its cell bodies. These results indicate that within a neuron RNA editing can recode genetic information in a region-specific manner.

## INTRODUCTION

In general, genetic information passes faithfully from DNA to RNA before being translated into proteins; however, there are exceptions. A variety of biochemical processes, collectively known as RNA editing, can alter it as it passes through RNA. The most common form of RNA editing in multicellular animals involves the hydrolytic deamination of adenosine to inosine (A→I), a nucleotide that is a biological mimic for guanosine (1). This process is catalyzed by the ADAR (adenosine deaminase that acts on RNA) family of enzymes and individual editing events regulate the functional properties of a wide variety of proteins, including ligand- and voltage-gated ion channels, neurotransmitter receptors and other messages that are vital for nervous system function (2). As with transcription, message recoding by A→I RNA editing is thought to take place exclusively within the nucleus, and this localization imposes constraints on the utility of the process. Nuclear RNA editing makes it difficult to regulate proteins differentially between cellular regions. However, evidence supporting the dogma that all recoding is nuclear is generally indirect and based on relatively few examples; only in humans, mice and flies has the overall extent of editing been determined co-transcriptionally (3–5).

ADAR’s localization and substrate requirements support the idea that A→I RNA recoding is nuclear. Mammals have two functional ADAR enzymes, ADAR1 and ADAR2 (6–9), and ADAR2 is the main message recoder (10). *Drosophila* expresses a single enzyme that is an ADAR2 ortholog (11). Both *Drosophila* and mammalian ADAR2s are localized to the nucleus and predominantly within the nucleolus (12–15). There are two main isoforms of mammalian ADAR1, termed p110 and p150, each driven by a different promoter (16–19). ADAR1 p110 is constitutively expressed and is localized to the nucleus like ADAR2. The expression of ADAR1 p150 is induced by interferon and the protein shuttles between the nucleus and cytoplasm due to the presence of both nuclear import and export signals (12,17,20–21). Although there is evidence that cytoplasmically localized ADAR1 P150 can edit messenger RNAs (mRNAs), the events are almost exclusively in Alu repeats and recoding out of the nucleus has never been demonstrated (22,23). ADAR substrates are thought to be nuclear as well. Most are within pre-mRNAs, made up of complex, higher order RNA folds that contain both exonic and intronic sequences (14,24–27). Because the substrates are essentially spliced out after transcription, their editing must occur within the nucleus. Not all substrates, however, rely on intronic sequence; for example, an entirely exonic structure drives editing within messages encoding the mammalian Kv1.1 channel (28). Thus, there is no definitive reason why mature mRNAs cannot be actively edited outside the nucleus at some sites. In addition, all data discussed thus far come from a limited group of organisms (mice, humans and flies). Other species may use the process differently.

The coleoid cephalopods use A→I editing to recode proteins at levels that are orders of magnitude higher than any other organism studied to date. The common market squid, for example, recodes about two-thirds of its neural messages by this mechanism and octopus and cuttlefish edit at similar frequencies (29–31). At present, the mechanistic differences that drive this high-level recoding are unknown. Squid and octopus genomes both encode ADAR1 and ADAR2 orthologs (29,32) and the substrate requirements of squid ADAR2 have been studied *in vitro* (33,34). Mature messages encoding a squid K^+^ channel and a Na^+^/K^+^ ATPase α subunit can be edited by squid ADAR2 showing that, in at least some cases, intronic sequence is not required to form suitable structures (33–35). Due to the sheer number of recoding events, editing in cephalopods has the potential to regulate a wide variety of physiological processes. How this potential is utilized is poorly understood. In this study, we ask whether editing can be deployed to regulate genetic information regionally within a neuron.

## MATERIALS AND METHODS

### Expression of sqADAR2a and sqADAR2b

The expression and purification of recombinant SqADAR2 proteins from *Pichia pastoris* has been described previously in detail (34,36,37). For expression in HEK-293T cells, the sqADAR2a and sqADAR2b ORFs (Open Reading Frames; previously reported in Palavicini *et al.* 2009) were cloned into the pcDNA3.1(−) expression vector using the NheI and ApaI restriction sites. These constructs had a Kozak sequence engineered before the start codon (GCCACC), a HIS tag at the N-terminus and a FLAG tag at the C-terminus; these tags have been shown previously to not affect enzymatic function. A FLAG-tagged rat ADAR2 (rADAR2) in pcDNA3.1 was kindly provided by Dr Marie Ohman from Stockholm University. All constructs were verified by direct sequencing. The oligonucleotides used for cloning are shown in Supplementary Table S1. HEK-293T cells were maintained in Dulbecco’s modified Eagle’s medium supplemented with 10% (v/v) fetal bovine serum, 1% penicillin–streptomycin solution, 1 mM sodium pyruvate and 2 mM glutamine. For each transfection, 300 × 10^3^ cells were seeded in a 35-mm dish and 48 h later were transfected with 1 μg of plasmid DNA. The empty pcDNA3.1(−) vector was transfected as a negative control. The Effectene Transfection Reagent kit was used according to protocol. Cells were analyzed 48 h post-transfection.

### Immunohistochemistry in HEK-293T cells

For immunofluorescence, 300 × 10^3^ HEK-293T cells were grown on glass coverslips coated with 0.1% poly-d-lysine 2 days before transfection. Plasmid DNA encoding SqADAR2a, SqADAR2b and rADAR2 was transfected as explained earlier. Forty-eight hours after transfection, cells were washed with 1× phosphate buffered saline (PBS) and fixed with freshly made 4% paraformaldehyde–PBS for 15 min on ice, permeabilized in 0.1% Triton X-100 in PBS for 15 min and blocked with 10% normal donkey serum/1% bovine serum albumin solution for 10 min at room temperature. Cells were then incubated with an α-FLAG (1:500, Sigma, cat. # F3165) primary antibody overnight at 4°C and then incubated with an Alexa Fluor 546-labeled goat anti-mouse secondary antibody (1:800) for 1 h at room temperature. Hoechst 33342 dye (1:10 000; Thermo Fisher, cat. # 62249) in PBS was added to the cells for nuclear visualization. Images were acquired using a Nikon A1R confocal imaging system. For analysis, a total of 200 cells for each experiment were manually counted using the Fiji ImageJ software.

### Preparation of total proteins from HEK-293T cells

For the extraction of total protein, transfected cells were washed with 1× PBS and centrifuged at 200 × *g* for 5 min at 4°C. Samples were then lysed with 500 μl lysis buffer (500 mM NaCl, 50 mM HEPES, pH 7.4, 1% Triton X-100 and 10% glycerol), supplemented with 5 mM dithiothreitol, 0.5 mM phenylmethylsulfonyl fluoride and 1× Halt Protease Inhibitor Cocktail (Thermo Scientific) and centrifuged at 2000 × *g* for 20 min at 4°C. Total protein concentration was quantified using the Precision Red Advanced Protein Assay reagent according to protocol and samples were used for western blot.

### Squid collection, dissection and homogenization

Specimens of the squid *Doryteuthis pealeii* were collected from the Marine Biological Laboratory, Woods Hole, MA. The giant fiber lobe (GFL), stellate ganglion (SG), optic lobe (OL), giant axon (GA), small nerves, heart, gill and skin epithelium tissues were manually dissected from adult males. For axoplasm preparation, axons were dissected in Ca^2+^-free artificial seawater and the axoplasm was extruded from an 8–10 cm section of GA with a glass capillary on Parafilm. Immediately after dissection, total protein was extracted from all tissues by homogenizing in lysis buffer [1× tris-buffered saline, 0.1% sodium dodecyl sulfate (SDS), 10 mM β-mercaptoethanol, 5% Tween 20 and 10 mM ethylenediaminetetraacetic acid (EDTA)]. Lysates were spun at 13 000 rpm at 4°C for 5 min. Supernatant was quantified with Precision Red Advanced Protein Assay reagent according to protocol. For the cellular fractionation experiments, cells were fractionated into cytoplasmic and nuclear compartments with the NE-PER extraction kit (Thermo Fisher) according to protocol.

### Production of sqADAR2 rabbit antibodies

Antibodies were raised in rabbits to the sequence (C)HGQDVETGDRHPNRKARGQ found in both sqADAR2 isoforms. This corresponds to residues 426–444 of the sqADAR2b amino acid sequence with an exogenous cysteine added at the N-terminal. The peptide containing this sequence was synthesized commercially at 97.65% purity with N-terminal acetylation and C-terminal amidation (Pi Proteomics LLC). The peptide was covalently conjugated to maleimide-modified keyhole limpet hemocyanin (Imject, Pierce Biotechnology) according to the manufacturer’s instructions and emulsified in Freund’s complete (primary injection) or incomplete adjuvant (boost injections) at a final concentration of 200 μg KLH-peptide conjugate per ml. To immunize, rabbits were initially injected with 0.8 ml of the final adjuvant mixture, followed by boost injections every month. Blood collections were done 2 weeks after each boost and resultant antibodies purified from the antisera by affinity chromatography on peptide-linked columns (SulfoLink, Pierce Biotechnology) according to the manufacturer’s protocol.

*Antibody specificity*: Three independent approaches were used to assess the specificity of the sqADAR2 antibodies. First, western blots of HEK cell extracts exogenously expressing sqADAR2a, sqADAR2b, rADAR2 or empty vector transfected cells were performed to determine whether bands of the expected size for sqADAR2 isoforms were expressed only in extracts from sqADAR2 transfected cells. In addition, immunofluorescence visualizations of ADAR2 proteins in these transfected cells were assessed. Finally, peptide pre-blocked sqADAR2 antibody controls were used in all experiments with cells transfected with ADAR2 cDNA and in squid neuronal tissues to assess specificity of results. As discussed later (see the ‘Results’ section), all three tests demonstrated a high degree of specificity of the anti-sqADAR2 antibodies.

### Western blots

For total protein expression from transient transfections, 12 μg of lysates from cells transfected with either sqADAR2a or pcDNA3.1(−) empty vector and 3 μg of lysates from cells transfected with either sqADAR2b or rADAR2 were used. For determining expression of sqADAR2 in endogenous squid tissues and for cellular fractionation experiments, 50 μg total protein was loaded for all tissue extracts. Recombinant sqADAR2a and sqADAR2b purified from *P. pastoris* were loaded as control. All samples were run on a 4–20% (w/v) gradient gel, transferred onto polyvinylidene difluoride membranes, followed by blocking with SuperBlock T20 Blocking Buffer. Membranes were probed with α-FLAG at 1:3000 (Sigma), α-sqADAR2 at 1:1000, α-tubulin at 1:4000 or α-HIS3 at 1:4000 as primary antibodies overnight at 4°C. Membranes were then probed with either IRDye 680RD goat anti-rabbit (1:15 000) or IRDye 680RD goat anti-mouse (1:15 000) secondary antibody for 1 h at room temperature. For blocking controls, membranes were incubated with 1:1 anti-sqADAR antibody and the peptide matching the sequence of the epitope. All membranes were imaged using the Odyssey Fc imaging system according to manufacturer’s instructions.

### Immunofluorescence visualization of the cellular expression of sqADAR2

Isolated *D. pealeii* SG, OL and GA were placed in a fixative of 4% paraformaldehyde (Electron Microscope Services, using their 20% prepared solution) in artificial seawater for 2 h at room temperature. Fixed tissues were then washed three times in PBS, allowing 30 min between washes. Following incubation overnight at 4°C in a cryoprotectant solution of 30% sucrose in PBS, the tissues were mounted on a cryostat chuck in Neg-50 media, frozen and then cryosectioned at either 50 μm (OL) or 30 μm (SG, GA). Each section was immediately applied to SuperFrost Plus slides (Thermo Fisher) at room temperature. These specimens were stored at −20°C until used for ADAR2 immunolocalization studies.

For immunofluorescence studies, the following protocol was used: Slide-mounted sections were washed three times for 10 min in PBS in a Coplin jar and then drained and placed horizontally in a humidified chamber. 0.2 ml of a solution of 0.3% Triton X-100, 4% normal goat serum and 2% bovine gamma globulin in PBS was pipetted on the slide surface for 1 h to permeabilize the specimen and to reduce non-specific antibody binding. Then for each slide, the ADAR2 antibody (at 0.4 mg/ml) was diluted 100× in 0.2 ml of the permeabilization solution, carefully applied to the slide near the mounted specimen and the slide overlaid with a small Parafilm coverslip. The humidified chamber was then covered and the slides incubated with the antibody overnight at room temperature. The next day the primary antibody solutions were drained from the slides and the slides washed three times for 10 min in PBS to remove residual primary antibody. Secondary fluorescent antibody (Alexa Fluor 565, Molecular Probes) was applied at 100× dilution in permeabilization solution for 2 h as described earlier for the primary antibody. Following washing three times in PBS, the slides were briefly immersed in distilled water and allowed to dry in the dark. Finally, the slides were coverslipped in Vectashield antifade mounting medium containing 4′,6-diamidino-2-phenylindole (DAPI; Vector Laboratories) and To-Pro-3 (Molecular Probes) to stain nuclei at short (405 μm) and long (647 μm) excitation wavelengths, respectively.

*Pre-blocked antibody controls*: To control for non-specific absorption of the primary antibody, a section on a separate slide was incubated with ADAR2 antibody that had previously been combined with the peptide antigen used to generate it (see the ‘Production of sqADAR2 rabbit antibodies’ section). Thus, pre-blocking was achieved by mixing the ADAR2 antibody with a 50–100-fold molar excess of the ADAR2 peptide for 3 h at an antibody dilution of 10×. Following incubation, the pre-blocked control was then diluted to the final 100× dilution of the unblocked antibody and applied as described earlier.

Imaging of immunostained slides was done using a Zeiss LSM 510 laser scanning confocal microscope controlled by ZEN (Zeiss, Inc.) software. Optical sections in z-series were made at optimal intervals at 1 Airy unit. Images for publication were processed using ZEN, ImageJ (various versions) and Corel PhotoPaint X7, and laid out and labeled using PowerPoint 2016.

### Differential editing analysis of whole transcriptomes from the squid giant axon and its cell bodies

To compare transcriptome-wide RNA editing between axons and cell bodies, four adult male specimens of the squid *D. pealeii* were collected from the Marine Biological Laboratory, Woods Hole, MA. The GFL and GA tissues were manually dissected as described earlier. Following dissection, axoplasm was extruded from the GA sample and total RNA was extracted from both axoplasm and GFL samples using the Trizol reagent (Sigma Chemicals, St Louis, MO). The RNA-Seq libraries for all the samples were prepared using the TruSeq Stranded mRNA Sample Prep Kit, as described by the manufacturer (Illumina, cat. # RS-122-2103), and were sequenced by Illumina sequencing at the University of Chicago Genomic Facility using two lanes of the Illumina HiSeq 4000 instrument, generating 85–96 million 100-nt paired-end reads (Supplementary Table S2). RNA sequencing data are available at the Sequence Read Archive, accession PRJNA596281.

### Gene expression analysis

Transcript abundance was calculated using Kallisto ‘quant’ function, with 50 bootstrap iterations (PMID: 27043002), and a reference transcriptome of 11 966 previously assembled squid transcripts (31). To study differential expression between the GA and the GFL, we calculated log_2_(fold change) using transcript abundance estimates averaged over the four biological replicates. To look for functional enrichment, transcripts were first mapped to human genes using BlastX, with an e-value cutoff of 1e−6 (PMID: 2231712), and then ranked by expression fold change, in ascending or descending order. The ranked lists were assessed by Gorilla (38). ADAR differential expression was assessed and visualized using Sleuth (39).

### Differential editing analysis

To search for differential editing between the squid GA and the GFL, we first aligned the reads to the 11 966 previously assembled squid transcripts using Bowtie2 with local alignment configuration and default parameters (31). Editing was quantified for a list of previously known editing sites (31) using the REDItools command REDItoolsKnown with the following parameters: -v 0 -n 0.001 -c 0 -t 2 -q −30 -m 40 (40). For this analysis, we considered only editing sites covered by at least 20 reads in each of the eight samples. Sites with significant differential editing were identified using two one-tailed *t*-tests with a Benjamini–Hochberg multiple-testing correction (separately for each of the one-tailed tests; false discovery rate (FDR) ≤ 0.1).

### Non-specific RNA editing assay

Radiolabeled double-stranded RNA (dsRNA) substrate synthesis and non-specific editing assays were performed and analyzed as described previously (33,34). In general, a 711-bp dsRNA substrate was derived from the squid Na^+^ channel GFLN1 (GenBank AN L19979.1; nucleotides 2111–2808), amplified using primers tagged with the T7 promoter sequence (both primers) and radiolabeled by including [α-^32^P]-ATP in the transcription reaction. Squid tissues were manually dissected from adult males and total protein was extracted by homogenizing in lysis buffer (1× TBS, 0.1% SDS, 10 mM β-mercaptoethanol, 5% Tween 20 and 10 mM EDTA). Insoluble material was pelleted by centrifugation at 13 000 rpm at 4°C for 5 min and the protein concentration of the supernatant was quantified with Precision Red Advanced Protein Assay reagent according to protocol. For assays, 10 μg of total squid tissue extracts was incubated with 0.5 pM radiolabeled dsRNA substrate for 2 h at 35°C in Q140 (10 mM Tris–HCl, pH 7.9, 140 mM KCl, 10 mM NaCl, 20% glycerol) supplemented with 5 mM DTT, 0.5 mM PMSF and 0.5 μg/μl tRNA. As a positive control, recombinant sqADAR2b was added to the assay to 10 nM and water was used as a negative control. After incubation, dsRNA was digested using P1 nuclease, and the nucleoside monophosphates were spotted on a Polygram CEL 300 UV254 thin-layer chromatography plate (Macherey-Nagel) and separated using saturated (NH_4_)_2_SO_4_, 0.1 M sodium acetate (pH 6.0) and isopropanol (79:19:2, by volume) as the solvent. Plates were then dried, exposed and scanned using a Typhoon™ FLA 7000 phosphorimager.

### Specific RNA editing assay using axoplasm

Site-specific *in vitro* editing reactions were set up using squid axoplasm and a fragment of the SqKv1A message (U50543.1; nt 43–494) subcloned into the BamHI and EcoRI sites of the pUC19 vector and *in vitro* transcribed using T7 RNA polymerase (T7 mScript, CellScript). PCR primer sites were appended to each end along with a 5′ T7 promoter sequence. The full insert sequence is given in Supplementary Table S1. The axoplasm was obtained from the GA of *D. pealeii* dissected as described earlier. The axoplasm was mixed with an equal volume of 2× Q150 (30% glycerol, 0.3 M potassium glutamate, 20 mM Tris–HCl, pH 7.9, 2 mM DTT and 2 mM PMSF) on ice. Prior to the reaction, RNA substrate was refolded in a thermocycler (5.5 min at 95°C, a ramp rate of 2°C/s to 85°C, 30 s at 85°C, a ramp rate of 0.1°C/s to 25°C and 5 min at 25°C). Two microliters of axoplasm extract were added to a 20 μl reaction containing 2 fmol refolded target RNA (SqKv1A), 5 mM DTT, 0.5 mM PMSF, 1 μg tRNA and 10 μl 2× Q150. The reaction was incubated at 25°C for 2 h and RNA was purified via phenol–chloroform extraction, chloroform extraction and ethanol precipitation. cDNA was synthesized from the RNA using Superscript III Reverse Transcriptase and the reverse PCR primer and then used as a template for PCR amplification. The resulting amplicons were gel purified (Qiagen MiniElute kit) and Illumina adapters (Supplementary Table S1) were ligated on prior to MiSeq-Illumina sequencing at the Keck DNA sequencing facility of the Bay Paul Center of the Marine Biological Laboratory.

MiSeq reads were aligned to the reference SqKv1A seq using Bowtie2 with local configuration and default parameters (41). Editing analysis was performed by processing the aligned reads using the mpileup function in the SAMtools package, filtering out base calls with Phred quality (*Q*) <30 (42). From the processed sequences, we determined the probability that observed A to G events were caused by random error using a binomial distribution with the Benjamini–Hochberg multiple-testing correction. Sequencing error probability can be considered to be 0.1% for bases with a *Q* > 30; we considered an editing event within a dataset to be real when the adjusted *P*-value was <0.001. Five replicates for experimental and control reactions were then averaged.

## RESULTS

### Transiently expressed SqADAR2s are in the cytoplasm of human cells

**Figure 1.**
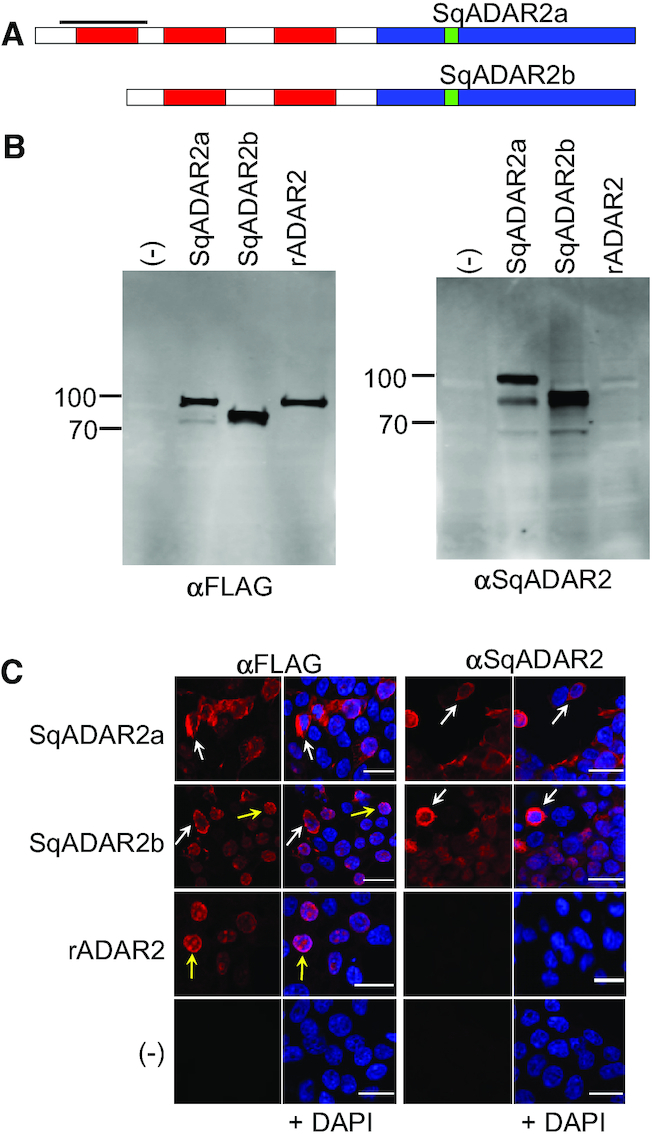
sqADAR2 enzyme is expressed in the cytoplasm in transiently transfected HEK-293T cells. (**A**) Schematic of the two ADAR2 isoforms expressed in squid (sqADAR2a and sqADAR2b). Deaminase domain is shown in blue, dsRBMs are shown in red and the epitope used for antisera development is shown in green. Bold line indicates the extra dsRBM found exclusively in sqADAR2a. (**B**) Western blot analysis of expression of sqADAR2a, sqADAR2b, rADAR2 and pcDNA3.1(−) empty vector after transient transfection in HEK-293T cells using either α-FLAG or α-sqADAR2. Membranes were imaged using a LI-COR odyssey infrared imaging system. Full-length sqADAR2a is 89 kDa, sqADAR2b is 71 kDa and rADAR2 is 83 kDa. No signal was detected in cells transfected with pcDNA3.1 empty vector; *n* = 2. (**C**) Immunostaining of full-length recombinant proteins after transient transfection in HEK-293T cells. Images are 100× and scale bars = 19 μm. Yellow arrows point to clear examples of cytoplasmic expression of both sqADAR2a and sqADAR2b enzymes. Cells were visualized using a Nikon A1R imaging system; *n* = 2.

To better understand the cell biology of high-level mRNA recoding in squid, specific reagents needed to be generated. In both mammals and *Drosophila*, ADAR2 isoforms are the main message recoders (10–11,43–44). Squid possesses two ADAR2 variants (SqADAR2a and SqADAR2b), generated through alternative splicing. An optional exon that encodes a double-stranded RNA binding motif (dsRBM) differentiates the two, yielding an atypical SqADAR2a with three dsRBMs in addition to the canonical SqADAR2b that contains two (Figure 1A) (33,34).

We generated antisera against an epitope in the enzyme’s deaminase domain that was shared by the two ADAR2 variants. As an initial test of the antisera’s specificity, FLAG epitope-tagged clones of SqADAR2a, SqADAR2b and rADAR2 were transiently transfected into HEK-293T cells and total protein extracts were processed for western blots (Figure 1B). As expected, when probed with an α-FLAG antibody, bands of the expected sizes were evident (89, 71, and 83 kDa for SqADAR2a, SqADAR2b and rADAR2, respectively). When the same samples were probed with the α-SqADAR2 antisera, only the sqADAR2s were evident. This too was expected because rADAR2 does not share the epitope used for antisera generation.

To further test the utility of the antisera, we immunostained HEK-293T cells transfected with the same constructs (Figure 1C). Confocal images of fixed cells confirmed the antisera’s specificity. When probed with either the α-FLAG antibody or the α-SqADAR2 antisera, no signal was detected in cells transfected with the pcDNA3.1(−) empty vector. Using the α-FLAG antibody, robust signals were present in all the transfected cells; however, the α-SqADAR2 antisera produced signals in only the SqADAR2a and SqADAR2b transfected cells.

Although the SqADAR2a and SqADAR2b signals were clear for both antibodies, their cellular localization was atypical. As reported by other groups, rADAR2 staining was mostly in the nucleus, and it showed puncta consistent with a nucleolar localization (12–13,45). This makes sense because the RNA structures that are required for recoding generally require both the intronic and exonic sequences found in pre-mRNAs prior to splicing, a process that generally takes place in the nucleus (13–14,25–26,46–47). Unlike rADAR2, both SqADAR2a and SqADAR2b showed clear cytoplasmic localization in many cells and nuclear localization in others (Figure 1C, white arrows and yellow arrows, respectively). Cytoplasmic localization was not evident in all cells, but it was frequent. Quantification of individual cells supported these conclusions: 87% of cells transfected with sqADAR2a that showed a signal showed some evidence of cytoplasmic staining, as did 81% of those transfected with sqADAR2b (*N* = 200 for each). By contrast, no cells transfected with rADAR2 showed cytoplasmic staining. Of course, human cells present a very different environment from squid cells with regard to ion composition, the precise machinery responsible for protein localization and other factors. Because these results could be due to an artifact of the expression system, the subcellular localization and distribution of SqADAR2 in endogenous squid nervous tissues was explored.

### SqADAR2 is expressed in the cytoplasm of squid neurons—I: western blots

**Figure 2.**
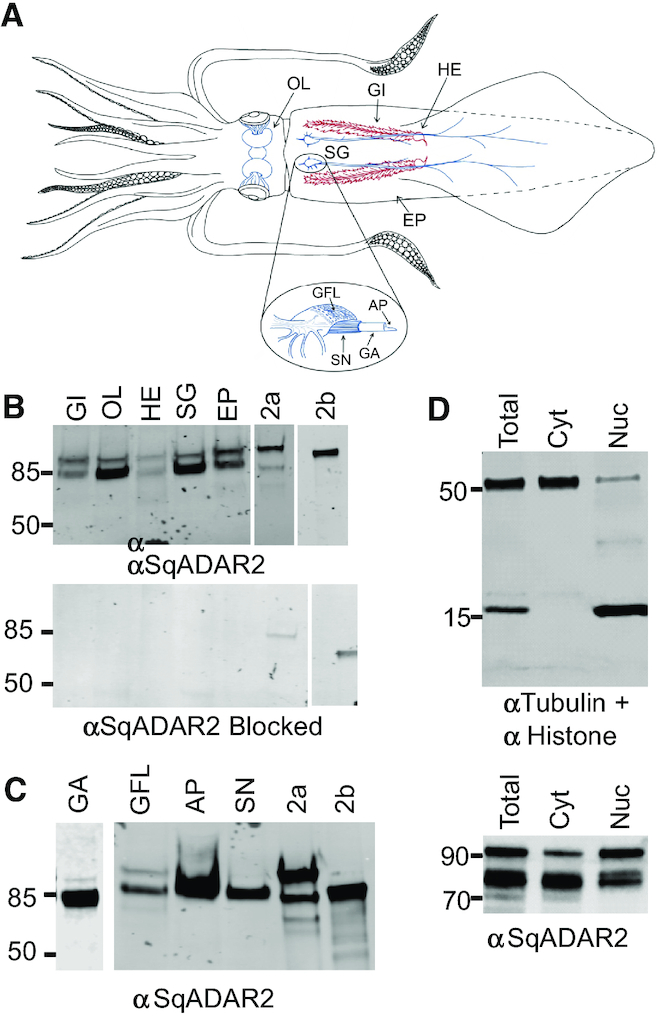
Extranuclear expression of sqADAR2 in squid tissue. (**A**) A schematic of squid anatomy showing the locations of tissue samples used in this study. OL = optic lobe of the CNS; GI = gill; HE = heart (branchial); SG = stellate ganglion of the PNS (note that the GFL is a part of the SG); EP = epithelium; GFL = giant fiber lobe; SN = small nerves (small diameter axons that form a mesh around the GA); GA = giant axon; AP = axoplasm. (**B**) Western blot analysis of nervous and non-nervous tissues extracted from *D. pealeii* and probed with α-sqADAR2 antibody (top panel). Blocked control is also shown (bottom panel). Purified sqADAR2 proteins (2a and 2b) were used as positive controls. (**C**) Western blot analysis of homogenates from different neuronal tissues extracted from *D. pealeii* and probed with α-sqADAR2 antibody. (**D**) Western blot analysis of whole somata samples from the squid SG after total, cytoplasmic and nuclear fractionation. Images were taken using a LI-COR odyssey infrared imaging system; *n* = 2. sqADAR2a (2a) is 89 kDa, sqADAR2b is 71 kDa, histone is 18 kDa and tubulin is 50 kDa. GI = gill; OL = optic lobe; HE = heart; SG = stellate ganglion; EP = skin epithelium; GA = giant axon; GFL = giant fiber lobe; AP = axoplasm; SN = small nerve fibers; 2a = sqADAR2a; 2b = sqADAR2b.

Western blots were used to examine the distribution and subcellular localization of SqADAR2a and SqADAR2b in squid tissues. To assess the specificity of the antisera, we tested homogenates from a variety of tissues and then blocked the signal by pre-incubating the antisera with the peptide against which it was raised (Figure 2A). Bands at the expected sizes for both sqADAR2s were evident in all tissues tested; however, they were most intense in nervous tissue [OL of the central nervous system (CNS) and the SG of the peripheral nervous system (PNS)]. Expression was similar in non-nervous tissue (gill, heart and epithelium); however, SqADAR2b was the more prominent of the two in nervous tissue. All bands were completely blocked by the peptide (lower panel).

To examine subcellular localization, we took advantage of a unique feature of squid anatomy. At ∼500 μm diameter and centimeter length, the GA from *D. pealeii* can be manually dissected. After dissection, the axoplasm can be extruded, separating it away from the plasma membrane and the enveloping Schwann cells. The GA is formed by the fusion of hundreds of small axons that emanate from the GFL neurons within the SG. The GFL neurons too can be isolated by manual dissection. Finally, the GA is surrounded by a mesh of parallel small diameter nerve fibers. Thus, axon, somata and even axoplasm can be analyzed in isolation.

Western blots of equivalent amounts of protein extracted from different neuronal tissues revealed strong signals in all nerve preparations (GA, SN and AP, Figure 2B). Signal was also present in the GFL (somata), albeit weaker. The nerve samples expressed SqADAR2b exclusively, whereas SqADAR2a could be seen in the somata. The fact that the signal was intense in the axoplasm means that the nerve signal cannot be derived from the Schwann cells. We next separated whole somata samples from the SG into nuclear and cytoplasmic fractions by centrifugation. Staining with α-tubulin and α-histone 2B antibodies confirmed the integrity of the separation (Figure 2C, upper panel). Processing these samples with the α-SqADAR2 antisera revealed bands for SqADAR2a and SqADAR2b in both the cytoplasm and the nucleus (Figure 2C, lower panel). These data suggested that the presence of cytoplasmic SqADAR2, first observed in HEK-293T cells, could also be observed in squid cells. Immunostaining of squid neurons was performed to further study this observation.

### SqADAR2 is expressed in the cytoplasm of squid neurons—II: immunostaining

**Figure 3.**
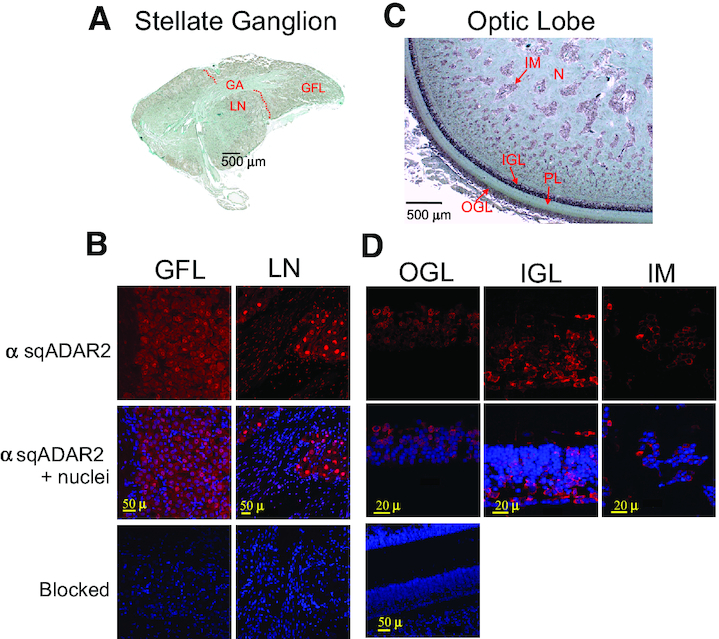
sqADAR2 is expressed in the cytoplasm of squid somata. (**A**) Sagittal section from the SG of *D. pealeii*. The GFL within the SG, large neurons (LN) outside the GFL and the squid GA are shown. A dashed red line delineates the border between the GFL and the rest of the SG. (**B**) Immunostaining of sqADAR2 proteins in both squid GFL and LN within the SG. Cells were stained with α-sqADAR2 primary antibody, while DAPI and To-Pro-3 were used for nuclear visualization. Blocked controls are also shown. (**C**) Sagittal section from the OL of *D. pealeii*. The structures comprising the OL including the outer granular layer (OGL), inner granular layer (IGL), plexiform layer, islands of the medulla (IM) and nuclei are shown. (**D**) Immunostaining of sqADAR2 proteins in neuronal cells within the squid OL. Cells were stained with α-sqADAR2 primary antibody, while DAPI and To-Pro-3 were used for nuclear visualization. Blocked controls are also shown. Cells were imaged using a Zeiss LSM 510 laser scanning confocal microscope.

To examine the cellular distribution of SqADAR in sections, we focused on two ganglia: the SG of the PNS and the OL of the CNS (Figure 3). Because the epitope used to generate our antisera is shared by SqADAR2a and SqADAR2b, we cannot distinguish between the two. The GFL, which is comprised of a monotype of neurons, forms a protrusion from the rest of the SG (48). The approximate border between the two is shown in a sagittal section through the SG (dashed line in Figure 3A). Outside of the GFL, the SG contains a more diverse array of neurons, and these include a zone of large diameter neurons near the border. The GA is formed by thousands of small diameter axons that leave GFL neurons and fuse (48,49). This structure can be seen as it exits the GFL and curves through the rest of the SG before leaving and extending along the musculature of the mantle.

SqADAR2 staining in both the GFL neurons and the large neurons of the SG was not uniform (Figure 3B). Whereas in both areas cytoplasmic staining is clear, nuclear staining varies. In the GFL, nuclear staining is generally weak or not evident. In the large neurons, it is intense in most cells, but not all. The variable nuclear staining is also evident in the smaller, elongated Schwann cell nuclei. As with the western blots, the signal is entirely blocked by pre-incubating the antisera with peptide. Thus, in both regions nuclear staining is variable and cytoplasmic staining is relatively uniform, yet most, if not all, neurons express SqADAR2.

In the central OL, SqADAR expression is even more variable. The optic nerve enters the OL in the plexiform layer of neuropil where it forms synaptic connections with the granular cell layers (Figure 3C) (50). The inner OL is comprised of large areas of neuropil surrounding islands of somata called the islands of the medulla. SqADAR staining in the granular cell layers is mostly cytoplasmic and varies greatly between neurons (Figure 3D). In fact, many cells lack SqADAR staining entirely, whereas others stain intensely. In the islands of the medulla, the pattern is very similar. Here, staining is entirely cytoplasmic and variable between cells. In the inner OL, there is no staining in the neuropil surrounding the islands of the medulla. As in the SG, the signal in all regions of the OL is blocked by pre-incubating the antisera with peptide. These data demonstrate that SqADAR2 is present in the cytoplasm of the somata.

### SqADAR2 is expressed in axons and synapses

**Figure 4.**
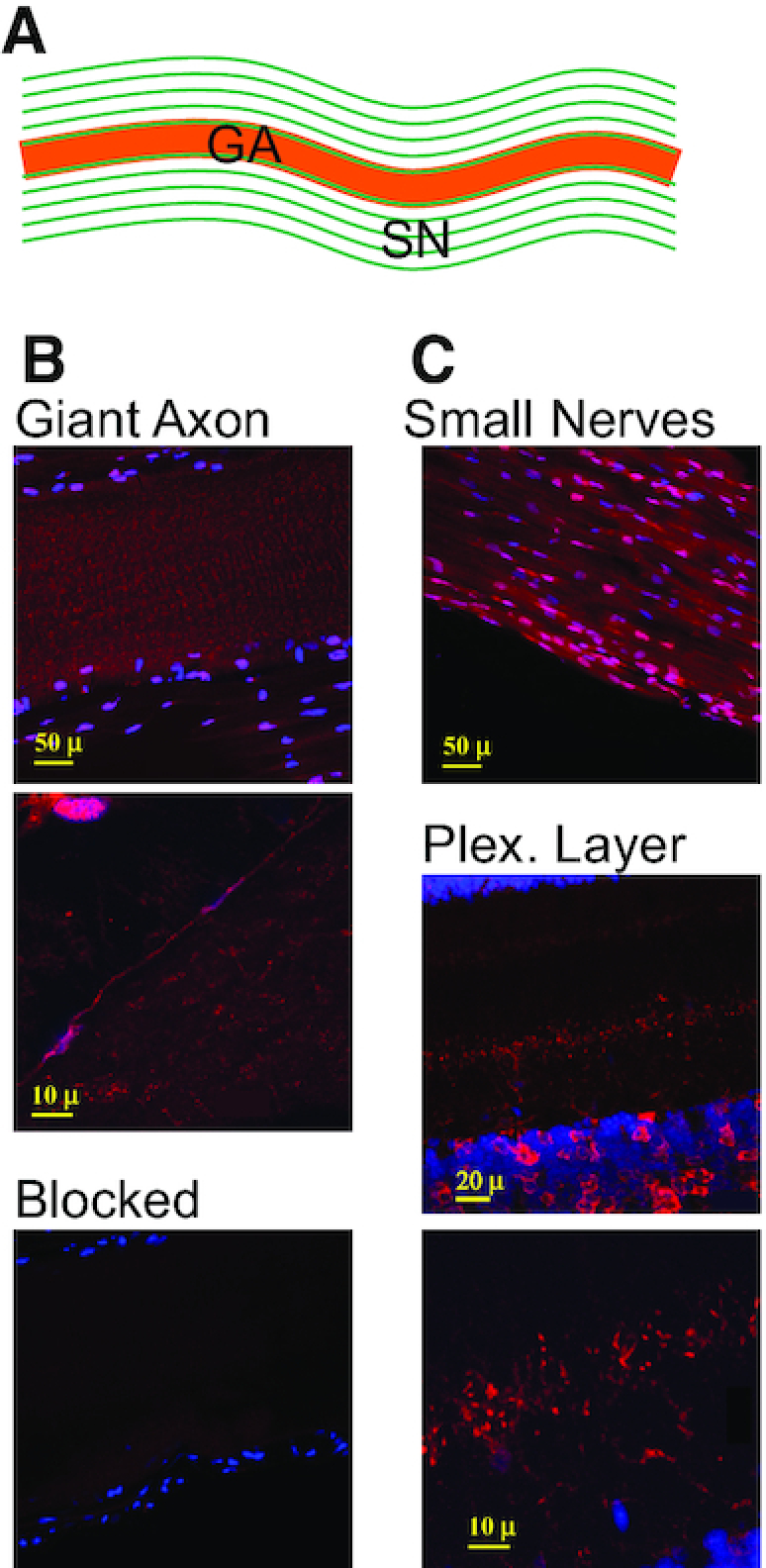
sqADAR2 is expressed in squid axons. (**A**) Diagram of the squid GA with its surrounding small nerve axons (SN) running in parallel. (**B**) Immunostaining of sqADAR2 proteins in squid GA (i). Higher magnification of same area is shown (ii). (**C**) Immunostaining of sqADAR2 proteins in the SN (i) and plexiform layer of the OL (ii). Higher magnification of same area or plexiform layer is shown (iii). Cells were stained with α-sqADAR2 primary antibody, while DAPI and To-Pro-3 were used for nuclear visualization. Blocked control is also shown (B, iii). Cells were imaged using a Zeiss LSM 510 laser scanning confocal microscope.

We also asked whether SqADAR2 can also be seen within axons and nerve terminals. The well-studied squid GA is a large diameter motor axon that exits the SG and innervates the musculature of the mantle (Figure 4A). It is surrounded by a sheath of small diameter axons that run in parallel. SqADAR2 staining is evident in longitudinal sections of the GA and the small nerves (Figure 4B and C). Higher magnification of the GA reveals that some of the staining occurs in discrete, intense puncta of <1 μM (Figure 4B, ii). Many of the puncta are lined up next to the cell membrane. The plexiform layer of the OL is primarily neuropil and contains two distinct bands of synaptic connections. SqADAR2 staining is evident in these bands, being most intense in the outermost band (Figure 4C, ii). A high-magnification image of this area shows that the staining is concentrated in distinct puncta (Figure 4C, iii); however, these, at ∼1–2 μM, are larger than those seen in the GA. All the signals in both the GA (Figure 4B, iii) and OL (Figure 3B) can be blocked by pre-incubating the antisera with peptide. Taken together, the western blot and immunostaining data indicate that there is robust SqADAR2 expression at several locations outside of the nucleus: (i) in the cytoplasm of the somata; (ii) in the axoplasm of axons; and (iii) at synapses. At the synapses, we cannot distinguish whether the staining is pre- or post-synaptic. The pattern is not exclusively cytoplasmic, as there is clearly SqADAR2 expression within nuclei as well. Furthermore, western blots show that it is the SqADAR2b isoform—not SqADAR2a—that is expressed in the axoplasm of the GA. These data lead to the question of whether mRNAs can be recoded outside of the nucleus.

### Squid axoplasm has an activity that can convert A→I

**Figure 5.**
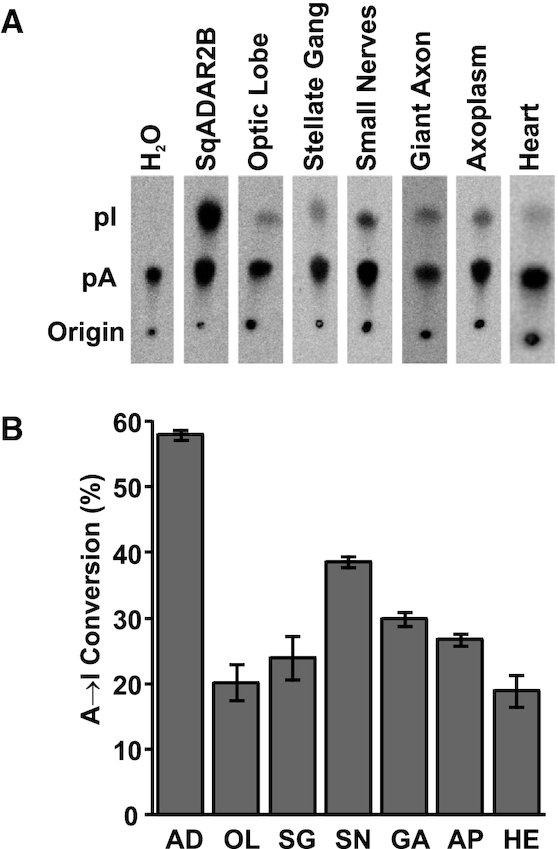
Axoplasm has A→I conversion activity. (**A**) Images of thin-layer chromatography plates used to separate radiolabeled adenosine and inosine from dsRNA editing assays with different tissues extracted from *D. pealeii*. Recombinant sqADAR2b was incubated as a positive control and assays with water were performed as negative controls. (**B**) Editing percentages were quantified based on A-to-I conversion; *n* = 3 ± standard error of the mean (SEM). AD = sqADAR2b; OL = optic lobe; SG = stellate ganglion; SN = small nerve fibers; GA = giant axon; AP = axoplasm; HE = heart. OL, SG and HE samples contain both nuclei and cytoplasm.

We next asked whether A→I conversion can occur in the axon. Two different experimental approaches were used as tests. In the first, tissue extracts from various regions were incubated with a perfect RNA duplex substrate in which all the adenosines were labeled with α-^32^P (Figure 5A). Following incubation, the reactions were digested with P1 nuclease to liberate nucleoside monophosphates and then run on thin-layer chromatography plates to assess the conversion of adenosine to inosine. As expected, a water (negative) control led to no A-to-I conversion and a recombinant SqADAR2b (positive) control led to a robust conversion of 58%. Conversion was evident in every tissue tested, ranging from 19% to 38% (Figure 5B). Notably, this includes small nerve, GA and axoplasm homogenates. Although equal amounts of lysate (based on protein concentration) were added to each reaction, it is difficult to directly compare activity between samples because they contain different proportions of cytoplasmic, nuclear and plasma membrane proteins and the dissection times to isolate them vary dramatically. The conclusion that nerve fibers and axoplasm are capable of A-to-I editing, however, is clear. Yet, the data do not show that the activity is site-specific and recodes codons.

### Squid axoplasm can edit naturally occurring sites in a K^+^ channel message

We next examined whether axoplasm can recode codons in mRNAs. To accomplish this, we assembled an RNA substrate containing 447-nt encoding part of SqKv1A, a voltage-dependent K^+^ channel (51). The SqKv1A message is known to be edited extensively in the GA system, and this substrate is particularly densely edited, containing 22 sites, half of which are edited at frequencies >10% (30–31,52). Unique PCR primer sites were tagged onto the ends of this substrate so that it could be distinguished from endogenous SqKv1A message. We incubated the substrate with freshly extruded axoplasm *in vitro*, or water in place of axoplasm as a control, for 2 h at 25°C. Following the reaction, total RNA was extracted, converted into cDNA and amplified by PCR. Amplicons were then sequenced on the Illumina MiSeq platform generating paired-end, 250-nt reads. Because the endogenous ADAR concentration in axoplasm is undoubtedly low compared to a standard *in vitro* editing reaction using recombinant ADAR, fractional editing would be low as well. However, with high enough sequence coverage, we reasoned that statistically significant editing events could be uncovered. Reads were then mapped back to the amplicon sequence and filtered for quality (statistics for this experiment are given in Supplementary Figure S1B).

We examined A→G conversions in all 126 adenosines in the substrate for five reactions treated with axoplasm from five different animals and five control reactions (Table 1). Two adenosines were edited at particularly high frequencies: positions 134 (0.78%) and 418 (10.33%) were edited at rates 78 and 258 times greater than the controls (individual editing percentages for each reaction are given in Supplementary Figure S1A). Furthermore, both sites, which recode positions in the channel’s tetramerization domain, are edited at high frequencies in squid. In a previous study, editing at position 134 (N45S) was shown to affect channel tetramerization (52). Position 418 (I140V) was not tested. In addition to these sites, three other positions showed significant A→G conversions: nt 139, 175 and 190. It is noteworthy that all five sites detected in the *in vitro* assay occur at sites that are naturally edited in the squid. However, 17 other sites that are edited *in vivo* were not edited in the *in vitro* assay. These data indicate that ADAR present in GA axoplasm has the capacity to recapitulate site-specific mRNA editing.

**Table 1.** Site-specific RNA editing activity in giant axon axoplasm

		Editing *in vitro*			
Editing *in vivo*		Control		Axoplasm	
Position	Percentage	Average	SEM	Average	SEM
44	27%	0.00	0.00	0.00	0.00
48	8%	0.02	0.00	0.02	0.00
63	20%	0.02	0.00	0.04	0.01
103	5%	0.01	0.00	0.01	0.00
107	48%	0.02	0.00	0.02	0.00
110	4%	0.02	0.00	0.02	0.00
127	52%	0.02	0.00	0.02	0.00
133	9%	0.02	0.00	0.04	0.01
134	69%	0.01	0.00	0.78	0.34
138	2%	0.04	0.00	0.05	0.00
139	15%	0.02	0.00	0.11	0.04
175	4%	0.02	0.00	0.08	0.03
190	2%	0.01	0.00	0.09	0.03
257	16%	0.02	0.00	0.02	0.00
259	60%	0.03	0.00	0.03	0.00
262	2%	0.03	0.00	0.03	0.00
361	2%	0.03	0.00	0.03	0.00
394	59%	0.02	0.00	0.04	0.01
395	21%	0.02	0.01	0.04	0.01
396	2%	0.01	0.00	0.01	0.00
418	94%	0.04	0.01	10.33	4.87
429	2%	0.05	0.00	0.05	0.00

A synthetic RNA substrate encoding a portion of the squid voltage-dependent K channel SqKv1A was incubated with extruded axoplasm. RNA was then extracted, converted to cDNA and amplified by PCR. The amplicon was then sequence with MiSeq. The table lists the location and editing percentages of adenosines that are naturally edited in the squid axon system and then the average and SEM of editing at the same positions in control and axoplasm reactions.

### Messages are edited more extensively in axons than cell bodies

The fact that SqADAR2 is expressed in the GA, and that axoplasm from the GA can recode specific codons, prompts the question of whether recoding patterns differ between different regions of a neuron. To test this idea, we compared transcriptome-wide RNA editing between axons and cell bodies. Four GA–GFL pairs were dissected from four adult, male specimens of *D. pealeii*. Axoplasm was extruded from each GA, and the GFL cell bodies were manually separated from the GA. RNA was then purified and used to prepare Illumina sequencing libraries from each sample. Thus, GFL samples contain RNAs from the nucleus and the cytoplasm of the somata, while the GA samples contain axon-specific RNA. Accordingly, it is reasonable to assume that the GA RNA is a subset of the GFL RNA.

The GA transcriptome is much less diverse than the GFL transcriptome. Most transcripts are depleted in the GA compared with the GFL (Supplementary Figure S2A and B), and only 350 of the transcripts account for 90% of the total expression in the GA [mean over four GA replicates = 349.75, standard deviation (SD) = 10.37], in comparison with ∼1400 transcripts accounting for 90% of GFL expression (mean over four GFL replicates = 1404.5, SD = 63.73; Supplementary Figure S2C). Gene ontology (GO) analyses (Supplementary Figure S3A) indicated that these are enriched in genes coding for proteins involved in RNA processing and metabolism, RNA translation and protein transport. Transcripts highly expressed in the GFL showed enrichment to neural processes such as synaptic signaling and ion channel activity, but to a lesser extent (Supplementary Figure S3B). It should also be noted that both SqADAR1 and SqADAR2 messages were barely detectable in the GA (Supplementary Figure S2D and E). Thus, the SqADAR2 protein that is detected in the GA is most likely translated in the cell bodies and then transported to the axon.

**Figure 6.**
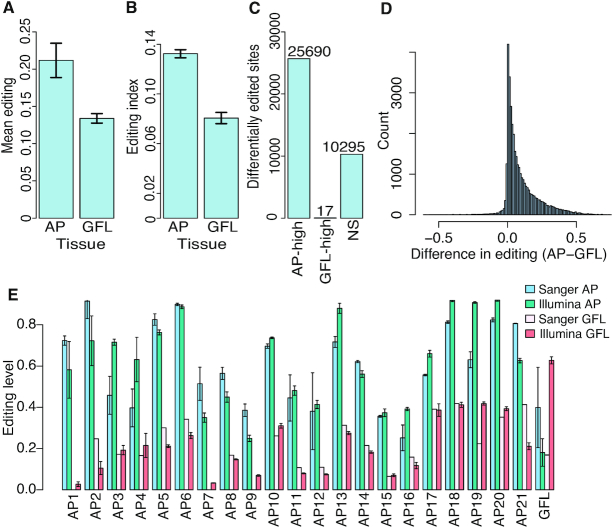
Editing is globally higher in GA versus GFL. (**A**) Mean editing levels over 36 002 editing sites in GA and GFL. Error bars represent SD over four replicates. (**B**) Editing index calculated using 36 002 editing sites in GA and GFL. Error bars represent SD over four replicates. (**C**) Number of sites with significantly higher editing in GA and in GFL and with no significant change in editing. (**D**) Histogram of the difference between editing in GA and editing in GFL in each site. Higher editing level in the GA is observed for 92% of the sites. (**E**) Sanger sequencing validation of differential editing sites. Error bars for the Illumina represent SEM over four replicates. Error bars for GA Sanger bars represent SEM over two replicates. No bars in the Sanger GFL lanes indicate that editing was not detected.

We then quantified RNA editing levels at the entire collection of sites determined in previous studies (30,31). Both the editing index (defined as the weighted average of editing levels over all editing sites) and the mean editing levels were consistently higher in the GAs than in the GFLs (Figure 6A). We next compared individual sites. Because our coverage was lower for these experiments than for the previous studies, only 36 002 sites out of the original set of 82 975 sites could be compared (see the ‘Materials and Methods’ section). Virtually all of these, 33 158 sites (92%) showed a higher editing level in the GA. Site-by-site statistical analysis resulted in 25 690 sites significantly more highly edited in the GA and 17 in the GFL, and 10 295 showed no significant difference (Figure 6C). Many of the differences in editing levels were substantial (Figure 6D). Editing increases in the GA across the board: 2289/2596 transcripts including sufficiently covered sites contain at least one differential GA editing site. There was no apparent enrichment of this effect for distinct functional pathways. Differential editing was then validated on a subset of sites. Seven amplicons from seven different messages were amplified by PCR from another GA–GFL pair from a new animal and the products were directly sequenced to quantify editing. These amplicons contained a total of 22 editing sites that were predicted by the Illumina data to be differentially edited, all but one of which were higher in the GA than the GFL. All 21 sites predicted to be more highly edited in the GA than the GFL were verified by Sanger sequencing in the new sample (Figure 6E). The one site predicted to be edited more extensively in the GFL was not corroborated in the Sanger data (GFL1). These data demonstrate that the RNA editing patterns differ between neuronal regions, with the majority of sites being more extensively edited in the GA.

## DISCUSSION

The idea that genetic information can be differentially edited within a cell is novel and extends our ideas about how a single blueprint of genetic information can give rise to spatial complexity. Such a process could fine-tune protein function to help meet the specific physiological demands of different cellular regions. Data from this paper suggest that region-specific RNA editing occurs in squid axons. We base this claim on the following findings: (i) SqADAR2 protein is present in the cytoplasm of nerve cell bodies in both the SG of the PNS and the OL of the CNS; (ii) axoplasm from the GA can catalyze the hydrolytic deamination of A→I in a perfect RNA duplex; (iii) axoplasm from the GA can catalyze site-specific RNA editing in a squid K^+^ channel substrate; and (iv) RNA editing at known sites is generally higher in the GA than its cell bodies. Editing almost certainly occurs in the nucleus as well. SqADAR2 is clearly present in many nuclei across the regions of the nervous system that were surveyed in this study. Therefore, based on these data we cannot exclude the possibility that all editing occurs in the nucleus and that edited messages bound to SqADAR2B get preferentially sorted to the axon. However, it seems unlikely that this mechanism could fully explain the observed differences in the editing patterns, given that axoplasm has active A→I conversion activity. For example, many messages in the axon contain some sites that are more highly edited than the cell bodies and others that are not. It is more likely that editing occurs in both the axon and the nucleus. This feature may be specific to cephalopods, suggesting that there may be fundamental differences about the editing process in this taxon.

On a mechanistic level, there are two possible explanations for SqADAR2B’s cytoplasmic localization. The first would be that following translation it gets imported into the nucleus inefficiently. The second would be that like vertebrate ADAR1 p150, it contains both nuclear import and export sequences and the equilibrium between these two processes determines its net distribution. The primary sequence of SqADAR2 does not offer clear clues that would help to distinguish between these possibilities. The nuclear localization signals of mammalian ADARs are not canonical (12–13,21) and there is no consensus motif in SqADAR2 either (34). SqADAR2 does not have a consensus nuclear export sequence as well; however, these sequences can be substantially degenerate and a motif at positions 306–315 (LNELRPGLKY) is a potential match (53). The balance between nuclear import and export has been shown to control subcellular localization in other proteins (21,54). Interestingly, within the nucleus ADAR’s nucleolar localization is driven by its dsRBM motifs that bind to rRNA (15). SqADAR2a has three dsRBMs and SqADAR2b has two; this could help explain why SqADAR2a was found predominantly within the nucleus while SqADAR2b was not.

Once out of the nucleus, the question remains as to how SqADAR2b protein gets into the axon following translation. Data from Supplementary Figure S2D and E shows that SqADAR2 message is primarily in the cell bodies; thus, the protein is almost certainly translated there as well. Given that the GA can extend for 25 cm or more, SqADAR like other proteins must be actively transported because diffusion rates would be too slow. Most cytosolic proteins are transported by a slow mechanism, and in the GA slow transport for tubulin, neurofilaments and other proteins is on the order of 0.1–4 mm/day (55,56). Fast transport in the GA, on the other hand, can move protein cargo at a rate of 5–40 cm/day (57). Having a better understanding of how SqADAR is moved along axons will give us a better understanding about how localized RNA editing might be used and regulated.

Differential editing was not balanced between the GA and the GFL. Over 70% of all the editing sites were edited significantly more highly in the GA than the GFL and 22/22 sites were validated using Sanger sequencing. Only 17 sites (<0.1%) were edited significantly more highly in the GFL and the one site examined by Sanger sequencing did not pass validation, leading to the possibility that the other 16 sites were false positives. All the rest showed no statistical difference between the regions. We should point out that we did not attempt to discover new editing sites in the GA, instead only looking at previously determined sites. The fact that essentially all differential sites were higher in the GA suggests that there may be new sites to discover. It also suggests a common mechanism underlying the phenomenon. Perhaps GA messages are in contact with SqADAR2 for longer durations, or there are other proteins in this region that help mediate contact. It was notable that immunostaining with the SqADAR2 antisera revealed clear puncta, suggesting that at least some of the enzyme is associated with complexes or granules. Beyond the mechanism, it is unclear why it would be advantageous to have generally higher levels of RNA editing in the GA.

Spatially regulated RNA editing in the GA system suggests that it imparts a functional advantage. If this is true, then the GA messages must be translated locally. Local translation in vertebrate axons has been well documented (58–60). In the squid GA, translation in isolated axoplasm has been observed for over 50 years (61–72) and a recent report suggests that membrane proteins can be synthesized locally as well (73). If so, do differentially edited sites affect protein function? In a past study, an editing event in messages encoding the α subunit of the squid Na^+^/K^+^ pump was examined on a mechanistic level (35). This edit recodes a highly conserved isoleucine in the seventh transmembrane span to a valine (I877V), causing a shift in the pump’s intrinsic voltage sensitivity by modifying the voltage-dependent release of Na^+^ ions to the external solution. The net effect of this change is to increase the pump’s forward transport rate. In this study, we revealed that the I877V edit occurs at a very low level in the GFL (2.8%) but at a substantial level in the GA (47%). Given that the I877V edit increases the pumping rate, there are some compelling reasons why this might be required in the GA. GFL cell bodies do not express voltage-dependent Na^+^ channels (74,75), while they are expressed at high densities in the GA (76). In addition, the membrane resistance in the GFL is close to 100 times higher than in the axon (77). Both these factors would lead to an increased Na^+^ leak in the GA and the consequent need to dispel it.

In this study, we examined differential editing between two regions: the GFL somata and the initial segment of the GA. SqADAR2 was seen, however, in the synaptic zone of the OL’s plexiform layer as well. It should be noted that the RNA isolated from the GA could have been in transit to other regions. The extent to which it is edited once reaching its destination, or whether it gets edited further after arriving, is unknown. Thus, it is likely that region-specific editing is more complicated in cephalopods than our data have uncovered. Does extranuclear recoding occur in other organisms? ADAR localization has only been examined in a few cases, under a small number of experimental conditions. Even in mammals, ADAR1 p150 is expressed in the cytoplasm and editable substrates occur within mature mRNAs. How this process is regulated in cephalopods should shed light on how RNA editing can be used to tune cellular physiology.

## Statement on the ethical treatment of animals

Although the use of cephalopods for research is not currently regulated in the USA, the Marine Biological Laboratory has implemented strict internal policies to ensure their ethical and humane treatment. All specimens of *D. pealeii* used in this study conformed to the Marine Biological Laboratory’s ‘Policy for the use of cephalopods for research and teaching’.

## Supplementary Material

gkaa172_Supplemental_Files
