# Supplementary material for: Spatially regulated editing of genetic information within a neuron
Source: Nucleic Acids Res. 2020 Mar 23;48(8):3999–4012. doi: 10.1093/nar/gkaa172 (PMC7192619; doi:10.1093/nar/gkaa172)
Supplement: gkaa172_Supplemental_Files [file gkaa172_supplemental_files.zip › Legends for Supporting Material.pdf]

Fig S1: Individual statistics for *in vitro* editing assays. (A) *In vitro* editing levels for Adenosines previously found to be edited *in vivo* in the mRNA of SqKv1A. Editing percentages and statistical significance are shown for each of the 5 animals studied and the 5 control reactions. (B) Summary of the MiSeq read and analysis metrics for each of the 5 animals studied and the 5 control reactions.

Fig S2: Differential gene expression in GA and GFL (A) histogram of transcript expression levels, measured in Log10(TPM+1), in GA and GFL samples. Error bars represent standard deviation over the four biological replicates. (B+C) Venn diagrams showing (B) lowly expressed genes (defined here as TPM<=1) in GA and in GFL and (C) highly expressed genes (defined here as TPM>=1000) in GA and in GFL. Expression of ADAR1 (D) and ADAR2 (E) in GA and GFL samples. Error bars represent 50 Kallisto bootstraps [74].

Fig S3: GO enrichment results of genes with a high relative expression in (A) GA vs. GFL and (B) GFL vs. GA.

Table S1. Sequences of oligonucleotides and constructs used in this study.

Table S2. Metrics on RNAseq from giant axon (GA) and giant fiber lobe (GFL) samples.
