# Supplementary material for: Spatially regulated editing of genetic information within a neuron
Source: Nucleic Acids Res. 2020 Mar 23;48(8):3999–4012. doi: 10.1093/nar/gkaa172 (PMC7192619; doi:10.1093/nar/gkaa172)
Supplement: gkaa172_Supplemental_Files [file gkaa172_supplemental_files.zip › Supporting Material.pdf]

Figure S1

A

	Axoplasm 1		Axoplasm 2		Axoplasm 3		Axoplasm 4		Axoplasm 5	
Position	Percentage	p-value	Percentage	p-value	Percentage	p-value	Percentage	p-value	Percentage	p-value
44	0.00	1.00	0.00	1.00	0.00	1.00	0.00	1.00	0.00	1.00
48	0.02	1.00	0.02	1.00	0.02	1.00	0.03	1.00	0.01	1.00
63	0.02	1.00	0.02	1.00	0.07	1.00	0.06	1.00	0.02	1.00
103	0.02	1.00	0.01	1.00	0.01	1.00	0.01	1.00	0.02	1.00
107	0.02	1.00	0.02	1.00	0.02	1.00	0.02	1.00	0.02	1.00
110	0.02	1.00	0.02	1.00	0.02	1.00	0.03	1.00	0.01	1.00
127	0.03	1.00	0.02	1.00	0.02	1.00	0.02	1.00	0.02	1.00
133	0.02	1.00	0.02	1.00	0.06	1.00	0.05	1.00	0.03	1.00
134	0.08	1.00	0.03	1.00	1.54	0.00	1.62	0.00	0.61	0.00
138	0.07	1.00	0.05	1.00	0.04	1.00	0.06	1.00	0.05	1.00
139	0.04	1.00	0.03	1.00	0.19	0.00	0.23	0.00	0.06	1.00
175	0.02	1.00	0.02	1.00	0.14	0.00	0.18	0.00	0.03	1.00
190	0.04	1.00	0.02	1.00	0.17	0.00	0.17	0.00	0.05	1.00
257	0.01	1.00	0.02	1.00	0.02	1.00	0.02	1.00	0.01	1.00
259	0.03	1.00	0.02	1.00	0.03	1.00	0.03	1.00	0.02	1.00
262	0.03	1.00	0.03	1.00	0.03	1.00	0.03	1.00	0.03	1.00
361	0.03	1.00	0.04	1.00	0.02	1.00	0.04	1.00	0.04	1.00
394	0.02	1.00	0.03	1.00	0.05	1.00	0.04	1.00	0.04	1.00
395	0.02	1.00	0.03	1.00	0.08	1.00	0.05	1.00	0.03	1.00
396	0.01	1.00	0.01	1.00	0.02	1.00	0.01	1.00	0.01	1.00
418	1.20	0.00	0.98	0.00	24.71	0.00	18.99	0.00	5.77	0.00
429	0.03	1.00	0.02	1.00	0.02	1.00	0.02	1.00	0.02	1.00

	Control 1		Control 2		Control 3		Control 4		Control 5	
Position	Percentage	p-value	Percentage	p-value	Percentage	p-value	Percentage	p-value	Percentage	p-value
44	0.00	1.00	0.00	1.00	0.00	1.00	0.00	1.00	0.00	1.00
48	0.02	1.00	0.02	1.00	0.02	1.00	0.02	1.00	0.02	1.00
63	0.02	1.00	0.02	1.00	0.02	1.00	0.02	1.00	0.02	1.00
103	0.01	1.00	0.01	1.00	0.02	1.00	0.01	1.00	0.01	1.00
107	0.02	1.00	0.02	1.00	0.02	1.00	0.02	1.00	0.01	1.00
110	0.02	1.00	0.02	1.00	0.02	1.00	0.02	1.00	0.01	1.00
127	0.02	1.00	0.02	1.00	0.02	1.00	0.02	1.00	0.02	1.00
133	0.02	1.00	0.02	1.00	0.03	1.00	0.02	1.00	0.02	1.00
134	0.01	1.00	0.02	1.00	0.02	1.00	0.02	1.00	0.01	1.00
138	0.04	1.00	0.05	1.00	0.04	1.00	0.04	1.00	0.04	1.00
139	0.02	1.00	0.02	1.00	0.02	1.00	0.02	1.00	0.02	1.00
175	0.02	1.00	0.02	1.00	0.02	1.00	0.02	1.00	0.01	1.00
190	0.02	1.00	0.02	1.00	0.02	1.00	0.01	1.00	0.01	1.00
257	0.02	1.00	0.02	1.00	0.02	1.00	0.01	1.00	0.01	1.00
259	0.03	1.00	0.03	1.00	0.04	1.00	0.03	1.00	0.01	1.00
262	0.04	1.00	0.03	1.00	0.04	1.00	0.04	1.00	0.02	1.00
361	0.04	1.00	0.03	1.00	0.03	1.00	0.03	1.00	0.02	1.00
394	0.02	1.00	0.03	1.00	0.02	1.00	0.02	1.00	0.02	1.00
395	0.01	1.00	0.02	1.00	0.04	1.00	0.04	1.00	0.02	1.00
396	0.01	1.00	0.01	1.00	0.01	1.00	0.01	1.00	0.01	1.00
418	0.03	1.00	0.05	1.00	0.06	1.00	0.05	1.00	0.02	1.00
429	0.02	1.00	0.02	1.00	0.02	1.00	0.03	1.00	0.02	1.00

B

	Axoplasm 1	Axoplasm 2	Axoplasm 3	Axoplasm 4	Axoplasm 5
Total Reads	153314	151884	161527	172121	138804
Paired Reads	153314	151884	161527	172121	138804
Concordantly 0 times	8133	7923	6275	7810	7642
Concordantly exactly 1 time	145094	143898	155048	163930	128735
Concordantly >1 times	87	63	204	381	2427
Aligment Rate	95.15%	95.24%	96.67%	96.11%	95.8%
Q>30	27683-143829	29995-142612	33714-153532	35640-162255	34432-126954

	Control 1	Control 2	Control 3	Control 4	Control 5
Total Reads	133888	149321	171320	158754	404058
Paired Reads	133888	149321	171320	158754	404058
Concordantly 0 times	4106	4906	7183	6180	22007
Concordantly exactly 1 time	129736	144313	163666	152380	375215
Concordantly >1 times	46	102	471	194	6836
Aligment Rate	97.38%	97.30%	96.79%	96.78%	95.94%
Q>30	26179-128621	24219-142819	31157-160323	29532-150916	135723-369207

Figure S2

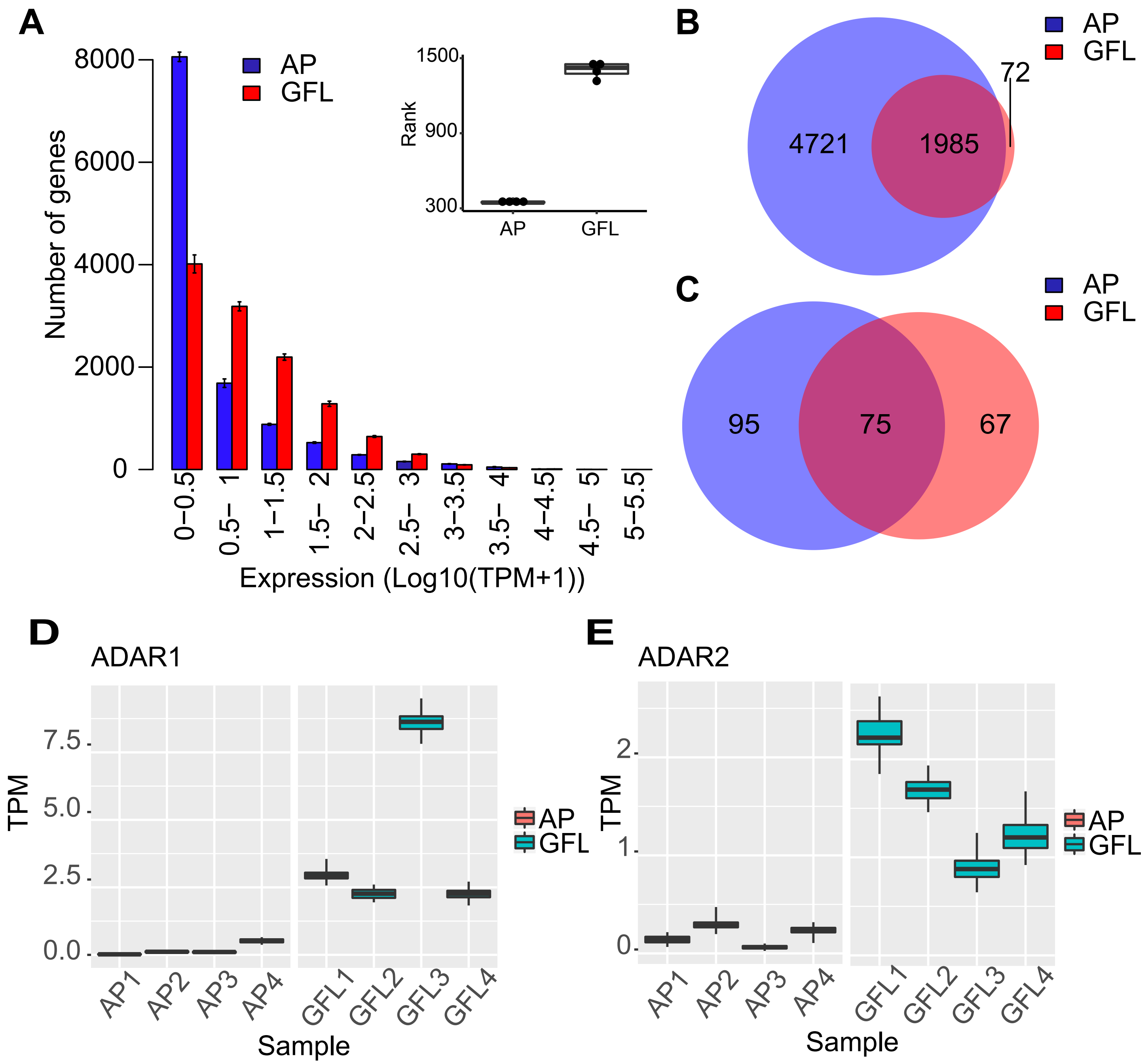
A
B
C
D
E
Number of genes
8000
6000
4000
2000
0
AP
GFL
0–0.5
0.5–1
1–1.5
1.5–2
2–2.5
2.5–3
3–3.5
3.5–4
4–4.5
4.5–5
5–5.5
Expression (Log10(TPM+1))
Rank
1500
900
300
4721
1985
72
95
75
67
ADAR1
ADAR2
TPM
7.5
5.0
2.5
0.0
2
1
0
AP1
AP2
AP3
AP4
GFL1
GFL2
GFL3
GFL4
Sample

Figure S3

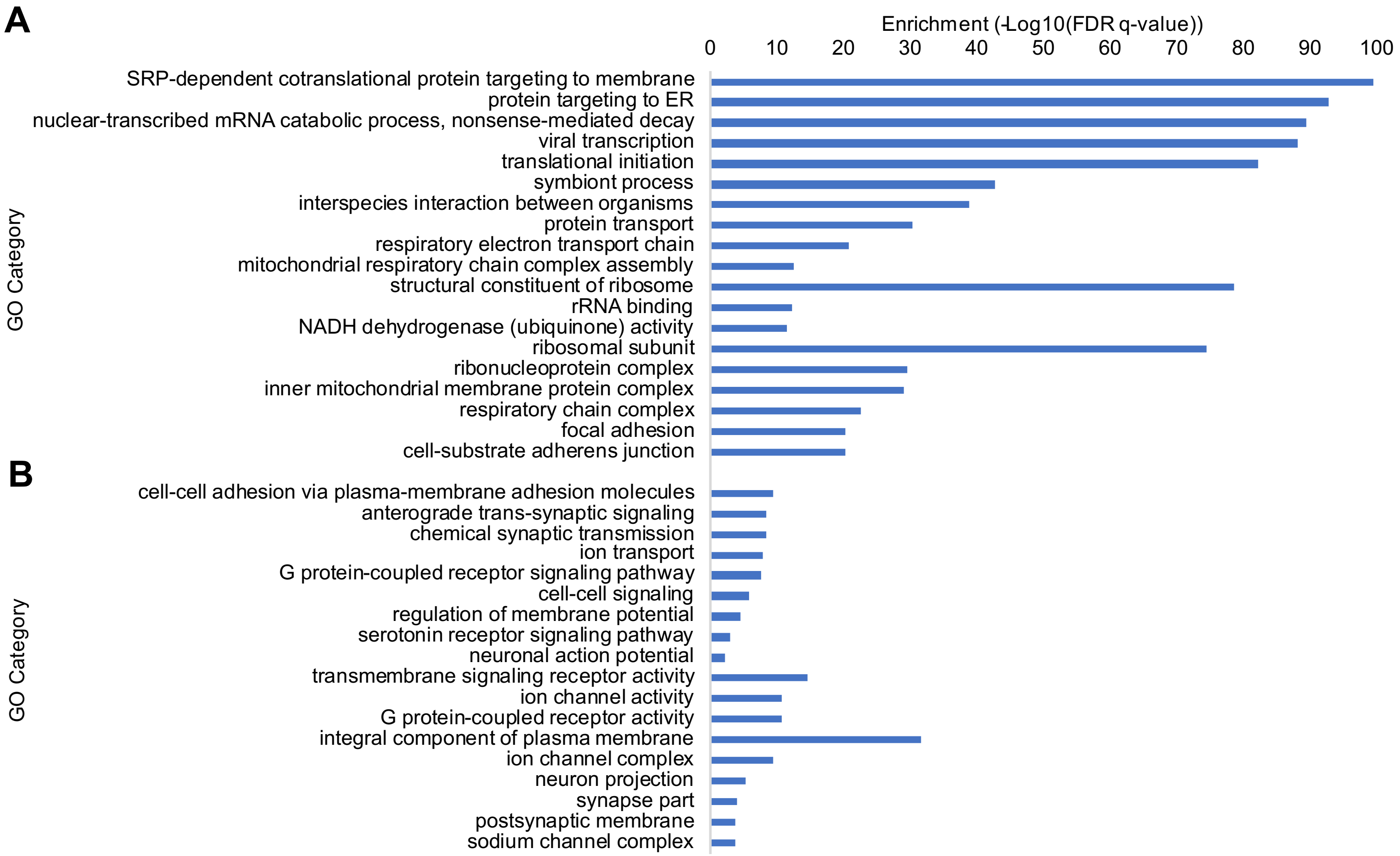

A
Enrichment (-Log10(FDR q-value))
0
10
20
30
40
50
60
70
80
90
100
GO Category
SRP-dependent cotranslational protein targeting to membrane
protein targeting to ER
nuclear-transcribed mRNA catabolic process, nonsense-mediated decay
viral transcription
translational initiation
symbiont process
interspecies interaction between organisms
protein transport
respiratory electron transport chain
mitochondrial respiratory chain complex assembly
structural constituent of ribosome
rRNA binding
NADH dehydrogenase (ubiquinone) activity
ribosomal subunit
ribonucleoprotein complex
inner mitochondrial membrane protein complex
respiratory chain complex
focal adhesion
cell-substrate adherens junction
B
GO Category
cell-cell adhesion via plasma-membrane adhesion molecules
anterograde trans-synaptic signaling
chemical synaptic transmission
ion transport
G protein-coupled receptor signaling pathway
cell-cell signaling
regulation of membrane potential
serotonin receptor signaling pathway
neuronal action potential
transmembrane signaling receptor activity
ion channel activity
G protein-coupled receptor activity
integral component of plasma membrane
ion channel complex
neuron projection
synapse part
postsynaptic membrane
sodium channel complex

Oligo/Construct Name	Sequence	Target Sequence	Description
NheI_HIS_sqADAR2	*GCTAGC*GCCACC**ATG<br>CACCATCATCATCACC<br>AC**CGGGACTTACAGG<br>CTAAAAA	4-23 NT sqADAR2 ORF	PCR forward primer for cloning sqADAR2a and sqADAR2b in pcDNA3.1(-). NheI restriction site is shown in italics. Kozak sequence is underlined. Start codon followed by the HIS6X tag is shown in bold.
Flag_sqADAR2_ApaI_R	T*GGGCCC***TCAGTCAT<br>CGTCGTCCTTGTAGTC<br>GAC**GACTAGTGCAGA<br>TGTTTCTG	2345-2364 NT sqADAR2 ORF	PCR reverse primer for cloning sqADAR2a and sqADAR2b in pcDNA3.1(-). ApaI restriction site is shown in italics. FLAG tag and stop codon are shown in bold.
SqKv1A *In Vitro* editing Substrate	TAATACGACTCACTAT<br>AGGGTGGTTGCCATA<br>AGCGGATCATCGGGA<br>GGAGAAACGGGGaCT<br>TAAAAAACCCGGATGA<br>AGAGAACAACCAGTTG<br>GATGCTGGGAGTGGT<br>TCTCTTCATaTGTaTAa<br>GCGGTCCGACCGTGT<br>TaTCATCaaCGTAaGC<br>GGACTTCGGTTTGAAA<br>CACAAACCCGTACACT<br>AaGCCAGTTTCCGGAT<br>aCGTTGTTGGGTAACC<br>CCAAAAAGCGAAACC<br>GCTATTATGACCCATA<br>CCGGAACGAATATTTT<br>TTTGaTaGGaATCGTC<br>CTAGTTTTGATGCCAT<br>CCTTTATTTTTACCACA<br>GTGGCGGGCGCCTCC<br>GGAGACCCCAAAATG<br>TGCCACTGGACATTTT<br>TCTTGAAGAGATCaGA<br>TTTTTCGAGCTCGGGG<br>AGGAGGTCATTGACaa<br>ATACCGTGCAGAAGA<br>GGGATTTaTCAAGGAA<br>GTaGAAAAACCCCTTC<br>CCGAGAACGAATTCCA<br>GCGACGTGTATGGCT<br>CTTGGTTGAGCACCC<br>GGATAGCGAGGCGAG<br>CGTTCCAAA	nt 43-494 ORF of SqKv1A	In blue is the T7 promoter sequence. In yellow are PCR primer tags. Lower case a's indicate the sites of naturally occurring RNA editing events
Illumina F MiSeq Adapter	AATGATACGGCGACC<br>ACCGAGATCTACACTC<br>TTTCCCTACACGACGC<br>TCTTCCGATCT		Universal F Illumina Adapter

Illumina R MiSeq Adapter	CAAGCAGAAGACGGC ATACGAGAT******GTG ACTGGAGTTCAGACGT GTGCTCTTCCGATCT		Universal R Illumina Adapter; *'s indicate index

Table S1. Oligonucleotides used for sqADAR2a and sqADAR2b cloning in HEK-293T cells.

Sample	Number of reads	Aligned reads	% Aligned reads
AP1	96822870	84690362	87.46
AP2	93299932	81475968	87.32
AP3	93246720	79917409	85.70
AP4	85963571	74954402	87.19
GFL1	93941535	74647335	79.46
GFL2	93114381	75188718	80.74
GFL3	90467954	72186913	79.79
GFL4	86841698	69348721	79.85

Supporting table 2. Metrics on RNAseq from axoplasm (AP) and giant fiber lobe (GFL) samples
